# Transcription Reprogramming during Root Nodule Development in *Medicago truncatula*

**DOI:** 10.1371/journal.pone.0016463

**Published:** 2011-01-27

**Authors:** Sandra Moreau, Marion Verdenaud, Thomas Ott, Sébastien Letort, Françoise de Billy, Andreas Niebel, Jérôme Gouzy, Fernanda de Carvalho-Niebel, Pascal Gamas

**Affiliations:** Laboratoire des Interactions Plantes Micro-organismes, Centre National de la Recherche Scientifique – Institut National de la Recherche Agronomique, Castanet-Tolosan, France; Ecole Normale Superieure, France

## Abstract

Many genes which are associated with root nodule development and activity in the model legume *Medicago truncatula* have been described. However information on precise stages of activation of these genes and their corresponding transcriptional regulators is often lacking. Whether these regulators are shared with other plant developmental programs also remains an open question. Here detailed microarray analyses have been used to study the transcriptome of root nodules induced by either wild type or mutant strains of *Sinorhizobium meliloti*. In this way we have defined eight major activation patterns in nodules and identified associated potential regulatory genes. We have shown that transcription reprogramming during consecutive stages of nodule differentiation occurs in four major phases, respectively associated with (i) early signalling events and/or bacterial infection; plant cell differentiation that is either (ii) independent or (iii) dependent on bacteroid differentiation; (iv) nitrogen fixation. Differential expression of several genes involved in cytokinin biosynthesis was observed in early symbiotic nodule zones, suggesting that cytokinin levels are actively controlled in this region. Taking advantage of databases recently developed for *M. truncatula*, we identified a small subset of gene expression regulators that were exclusively or predominantly expressed in nodules, whereas most other regulators were also activated under other conditions, and notably in response to abiotic or biotic stresses. We found evidence suggesting the activation of the jasmonate pathway in both wild type and mutant nodules, thus raising questions about the role of jasmonate during nodule development. Finally, quantitative RT-PCR was used to analyse the expression of a series of nodule regulator and marker genes at early symbiotic stages in roots and allowed us to distinguish several early stages of gene expression activation or repression.

## Introduction

The development of plant genomics during the last decade has led to an impressive accumulation of data on gene identification, notably through full genome sequencing and gene expression analyses using a variety of tools. This has led to a more detailed view on the genetic program(s) associated with complex processes and the possibility of defining both shared and specific components of various plant responses and developmental programs. One of the major challenges now faced by biologists is the identification of the regulators that orchestrate these programs, and the definition of the regulatory networks that they control or participate in.

Transcription factors (TFs) have attracted major interest, given the central role they play in regulating gene expression. They correspond to a large fraction of plant genes, representing more than 5% of the *Arabidopsis thaliana* genome [1], of which only a very small proportion has been molecularly or genetically characterized to date (about 10% in *A. thaliana*, [2] and less than 1% in legumes [3]). The considerable progress that has been made in the last few years in legume genomics, notably by sequencing the genome of three model species (*Lotus japonicus*, *Glycine max* [soybean] and *Medicago truncatula*) has allowed a general survey of legume TFs and their relation to other plant TFs [4]. Despite the fact that symbiotic nitrogen fixation (SNF) is a signature feature of legumes, no specific TF families have been assigned to this process so far.

SNF allows legumes to grow efficiently under nitrogen-limiting conditions, with very important agronomical and environmental benefits, due to their capacity to establish endosymbioses with soil bacteria collectively known as rhizobia [5], [6]. SNF involves mutual recognition of both partners through an exchange of molecular signals. Bacterial molecules responsible for the specific recognition of the rhizobial partner are called Nod factors (NF). These chito-lipo-oligosaccharides are able to trigger a number of plant responses including ion fluxes, calcium spiking, specific gene induction, root hair deformations and cortical cell divisions. Following the initial step of plant-bacteria recognition, rhizobial infection of root tissues occurs *via* tubular structures of plant origin, called infection threads, and is concomitant with inner cortical cell divisions leading to the formation of the specialised root organ known as the nodule. In addition to symbiotic NF, rhizobial cell wall-associated or secreted polysaccharide molecules [7] are also required for successful infection thread growth as far as the inner root tissues.

The root nodule is essential to provide rhizobia with both a carbon source and an appropriate cellular environment allowing the bacterial nitrogenase to fix atmospheric nitrogen. In temperate legumes, represented by the model legume *M. truncatula*, the nodule is a highly structured organ with indeterminate growth resulting from the activity of an apical meristematic region (also called zone 1) and the differentiation of both peripheral and internal tissues. The latter comprise an infection zone 2 where bacteria are released from infection threads and where coordinated differentiation of both plant and bacterial cells takes place. This leads to the formation of the zone 3 in which nitrogen fixation is carried out by terminally differentiated bacteroids. In several-week-old nodules, the degeneration of both symbionts initiates in the proximal region of zone 3, resulting in the formation of the senescence zone 4. Senescence can also be triggered prematurely by different stresses or defects in plant-bacteria recognition [8]. Furthermore, nodule and activity are tightly regulated by the plant and influenced by specific phytohormones. Thus jasmonic acid, ethylene, salicylic acid and abscisic acid have been reported to act as negative regulators, whereas cytokinins and auxins play positive roles in nodule initiation [5], [6], [9].

In the past two decades, important progress using genetic and molecular approaches has been made for the model legumes *L. japonicus* and *M. truncatula* in the identification of legume genes involved in the initial symbiotic stages associated with NF perception and transduction. The characterization of these genes indicates that NF perception involves receptor-like kinases and a transduction pathway through a specific calcium oscillation response that precedes early nodulin gene transcription by GRAS (NSP1 and 2) [10]–[13], and ERF (ERN1, 2 and 3) [14], [15] transcription factors. In addition, transcriptomic analyses in *M. truncatula* have led to the discovery and characterization of a number of genes preferentially expressed in nodules or in roots following inoculation with the microsymbiont *Sinorhizobium meliloti* [16]–[22]. Functional characterization of some of these genes, encoding the Zinc finger MtZPT2 [23], the CCAAT binding factor MtHAP2.1 [24]–[25] and the ERF EFD [26] have revealed important roles for these TFs during rhizobial infection and/or nodule organogenesis.

Transcriptomics approaches have been used in *M. truncatula* and *L. japonicus* to get a view of the range of genes associated with early or late stages of nodulation [16]–[22]. Earlier work has demonstrated that the simultaneous analysis of the transcriptome of wild type (WT) and symbiotic mutants is suitable to identify set of genes involved in specific symbiotic stages. In *M. truncatula*, the use of one bacterial and six plant mutants affected in early symbiotic genes allowed the identification of plant genes involved in early stages of NF signalling and bacterial infection [27]. More recently, cDNA microarrays representing 2,366 unigenes were used for an analysis of nodule development in the *M. truncatula* R108 line coupled with the independent analysis of a collection of 15 plant and bacterial mutants inducing non-functional, Fix^−^ nodules [28]. This study allowed the authors to conclude that two stages of gene expression reprogramming take place during nodule formation, the first at the immature nodule stage and the second when nodules reach maturity.

Here we present a large scale approach that aimed at identifying nodule-associated genes, and more specifically putative regulators of gene expression. By combining gene expression analyses using 70-mer oligonucleotide 16.4 K microarrays for both wild type symbionts and nodulation defective symbiotic mutants, we were able to identify and classify more than 3,400 differentially regulated genes and associated regulators. This has led us to define four distinct stages of transcription reprogramming throughout nodulation. In addition, a fifth set of genes are expressed during several stages or in different tissues and two final subsets of genes are associated with either senescence or stress/defence responses. We also used tools recently developed for data mining in *M. truncatula* to obtain complementary information on the expression pattern of these regulators in non-symbiotic conditions, as well as mid-scale quantitative RT-PCR analyses to investigate how expression in nodules relates to expression in roots.

## Results and Discussion

### WT and mutant strains of *Sinorhizobium meliloti* to analyse nodules at different developmental stages

**Figure 1:**
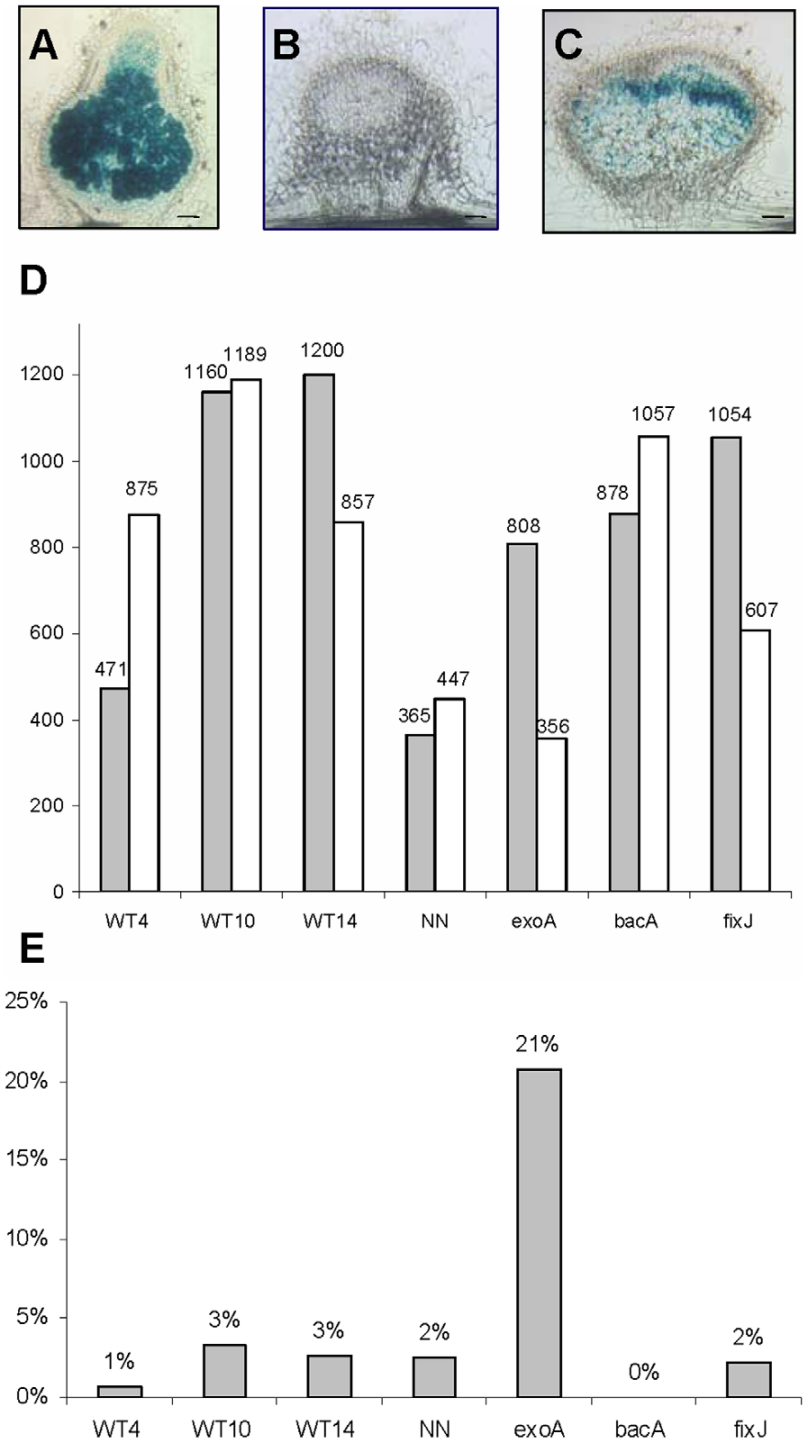
Transcriptome analysis of root nodules induced by wild-type and mutant strains of *Sinorhizobium meliloti*. A, B, C: 50 µm sections of representative 10-day-old nodules analysed in the experiment, respectively induced by wild-type (A), *exoA* (B) and *bacA* (C) *S. meliloti* strains containing a *hemA:lacZ* reporter; the blue color indicates the presence of bacteria revealed by beta-galactosidase activity. Bars = 100 µm. D: number of differentially regulated genes, in comparison to non-inoculated nitrogen-starved roots (adjusted Pval≤0.05 ratio ≥1.5), with white and grey columns representing and down- and up- regulated genes respectively. E: fraction of genes found to be up-regulated only in this nodule sample. WT4, 10, 14, NN correspond to nodules induced by wild-type *S. meliloti* 2011 at 4, 10, 14 and 16 days post-inoculation; NN nodules were treated for 2 days with 10 mM NH_4_NO_3_ before harvesting.

To gain a broad view on the transcriptional changes occurring during nodule development, the *M. truncatula* transcriptome at different nodulation stages was analysed using Mt16kOLI1Plus microarrays, which carry 16,470 *M. truncatula* probes [29], [30]. We compared a series of seven nodule samples from *M. truncatula* A17, following inoculation with WT or three mutant strains of *Sinorhizobium meliloti* impaired in symbiotic properties (Table 1). The nodule samples induced by WT *S. meliloti* strain 2011 were harvested either at an immature stage before the differentiation of the nitrogen-fixing zone 3 (4 dpi: WT4) or after the onset of nitrogen fixation (10 and 14 dpi: WT10 and WT14; Table 1 and Figure 1A). An additional sample, termed NN (for Nitrate-treated Nodules), was harvested at 16 dpi 2 days after the addition of 10 mM NH_4_NO_3_ in order to study the situation where nitrogen fixation has been turned off [31]. Three other nodule samples, harvested at 10 dpi, corresponded to nodules defective in different stages of development. These were obtained after inoculation with respective mutant strains of *S*. *meliloti*: *exoA* is defective in bacterial infection [32], *bacA* is affected in bacteroid differentiation [33] and *fixJ* is impaired in nitrogen fixation [34] (see Table 1).

**Table 1:** Seven *Medicago truncatula* nodule samples to examine different nodule developmental stages.

Nodule sample	Nodule formation	Nodule infection	Nodule differentiation	N-fixation	Nodule senescence
WT4	**+**	**+**	**−**	**−**	**−**
WT10	**+**	**+**	**+**	**+**	**−**
WT14	**+**	**+**	**+**	**+**	**−**
NN	**+**	**+**	**+**	**−**	**+**
*exoA*	**+**	**−**	**−**	**−**	**+**
*bacA*	**+**	**+**	**−**	**−**	**+**
*fixJ*	**+**	**+**	**+**	**−**	**+**

Wild type (WT) nodules were induced by WT *Sinorhizobium meliloti* and harvested at 4, 10, 14 (WT4, 10 and 14) and 16 dpi (NN). NN nodules were treated for two days with 10 mM NH_4_NO_3_. *exoA*, *bacA* and *fixJ* nodules were induced by the indicated mutant strain of *S. meliloti* and harvested at 10 dpi.

These seven nodule samples were compared to control samples comprising nitrogen-starved non-inoculated roots, grown under identical conditions (raw data available at ArrayExpress, accession number E-TABM-688). A threshold ratio of 1.5 with an adjusted P-value ≤0.05% was chosen to define genes as differentially regulated by comparison with control roots. This minimum ratio of 1.5 was based on our previous characterization of genes like *MtbHLH1*, which had induction ratios of below 2.0 on microarrays (1.79 at 4 dpi for *MtbHLH1*; Table S1) but which were clearly induced during nodulation according to quantitative RT-PCR ([19]) and promoter:GUS fusion analyses (data not shown).

**Figure 2:**
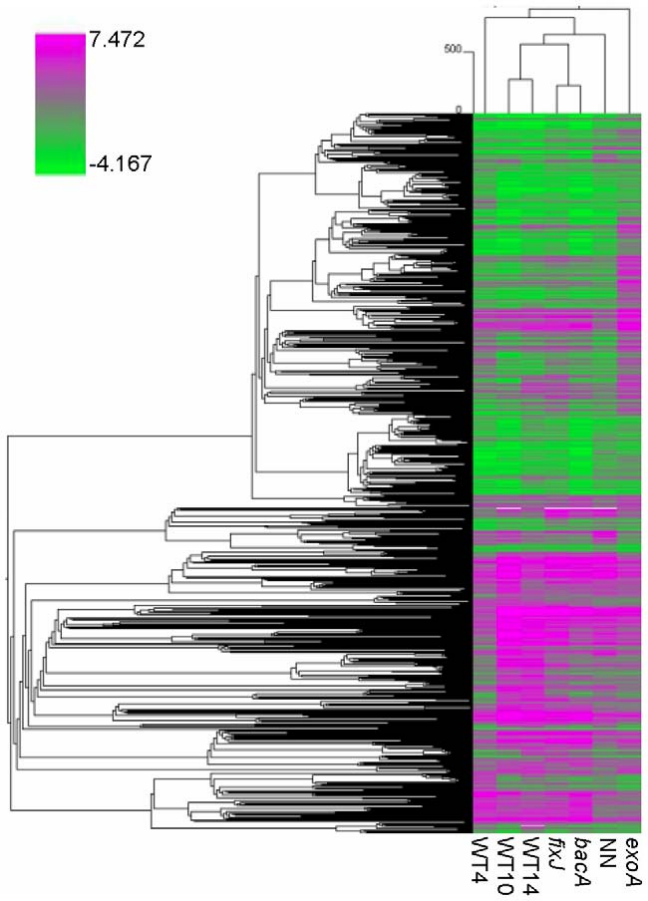
Hierarchical analysis of genes differentially regulated in *Medicago truncatula* nodules as compared to nitrogen-starved roots. These genes were identified by a comparison of non-inoculated roots and isolated nodule samples using Mt16KPlus microarrays The hierarchical analysis was carried out with Pearson correlation and average linkage. The green and pink colors indicate down- and up-regulated genes respectively, following the color scale shown on the left (with fold ratios indicated as log2 values).

A total of 3,437 gene probes were scored as differentially regulated (complete list provided in Table S1), corresponding to about 20% of the microarray gene probes. The largest number of differentially regulated genes was found for the *fixJ* and nitrogen–fixing nodules (WT10, WT14), whilst the smallest number corresponded to NH_4_NO_3_-treated NN nodules (Figure 1D). To evaluate the internal consistency of these results, we determined the fraction of genes up-regulated in several conditions vs. a single condition (Figure 1E); we also performed a hierarchical clustering analysis (Figure 2) and two by two comparisons (Table S2). All three methods indicated a very high overall consistency, with the WT10 and WT14 samples showing the closest profiles, as expected. Gene expression in the *exoA* nodules was the most different, with several gene probes showing opposite regulation as compared to the other samples (Table S2).

**Figure 3:**
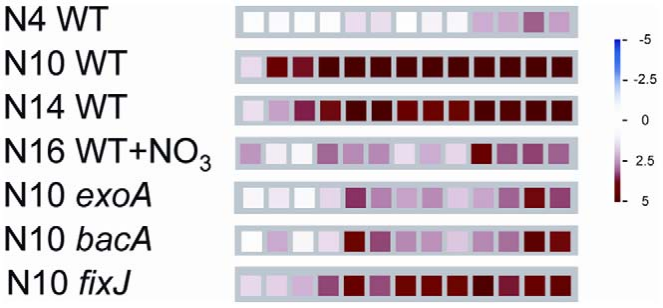
Expression of the leghemoglobin gene family in nodule samples (MapMan representation). Gene expression was analysed with Mt16KPlus microarrays, by comparison to nitrogen-starved uninoculated roots. Each square represents a different gene probe. Up-regulated genes are represented as red squares, following the color scale shown on the right (with fold ratios indicated as log2 values). WT4, 10, 14, NN correspond to nodules induced by wild-type *S. meliloti* 2011 at 4, 10, 14 and 16 days post-inoculation; NN nodules were treated for 2 days with 10 mM NH_4_NO_3_ before harvesting. *exoA*, *bacA* and *fixJ* correspond to nodules induced by indicated mutant strains of *S. meliloti* at 10 days post-inoculation.

An examination of the expression pattern of the leghemoglobin gene family revealed major increases for individual genes comparing immature to N-fixing nodules, with intermediate levels in nodules induced by mutant strains of *Rhizobium*, and decreased expression following ammonium nitrate addition (Figure 3) as previously shown [31]. The expression of three other marker genes was consistent with previously described results [27] in *fixJ* and *bacA* nodules: *MtCAM1* (MT007325) and *MtN31* (MT007417) are transcribed in *fixJ* but not in *bacA* nodules, whereas *MtLEC4* (MT007335) is expressed in both conditions. However, in contrast to the data reported in [27] we found that *MtLEC4* was expressed in *exoA* nodules. This can be explained by the fact that (i) isolated nodules instead of nodulated roots were analysed (ii) samples were harvested at an earlier time point than in [27], which is particularly important for *exoA* nodules that arrest growth several days after inoculation.

### Eight major activation patterns identified amongst nodule-associated genes

Our analyses allowed us to identify distinct expression profiles that could be classified into different groups or sub groups (Table 2). To assess the robustness of this classification, we compared our results to data from the *M. truncatula* gene atlas (MtGEA) [20] (http://mtgea.noble.org/v2/). These data include five conditions shared with this study (non inoculated roots, 4, 10, 14 and 16 dpi WT nodules) and come from independent samples generated in our laboratory but analysed with a different tool (Affymetrix chips). The expression pattern of 352 probes was examined and found qualitatively similar in 84% of the cases (Table S1). A substantial fraction (31%) of the 55 gene probes showing inconsistent patterns were scored as differentially expressed in only one of the nodule samples examined by 16KPlus microarrays. We therefore conclude that our classification is reliable, particularly when based on genes scored as differentially expressed in more than one condition.

**Table 2:** Definition of distinct expression classes amongst genes differentially regulated in *Medicago truncatula* nodules.

class designation	class or (sub)group description		number of probes	regulators of gene expression	fraction of up-regulated genes	fraction of activated regulators
1	down-regulated in nodules		1387	97		
2.1	zone1-2	strongest expression in immature nodules and no expression in *exoA* nodules	110	5	5%	3%
2.2	bacA	maximal expression in *bacA* nodules	87	6		
2.3	fixJ	maximal expression in *fixJ* nodules	124	5		
2.4	diff1	non induced in 4 dpi nodules, induced in 10 dpi and older nodulesincluding *bacA* nodules	347	27	17%	14%
	diff2	non induced in 4 dpi nodules, induced in 10 dpi and older nodulesbut not *bacA* nodules	188	12	9%	6%
2.5	fix+	mostly expressed in N-fixing nodules and expression decreased by nitrate addition	159	12	8%	6%
2.6	all*	expressed in most samples (not considering NN) but *exoA* nodules	95	5		
	all	expressed in most samples (not considering NN)	172	26		
2.7	NN	maximal expression in ammonium nitrate-treated nodules (16 dpi)	75	8	4%	4%
2.8	exo1	maximal (but not exclusive) expression in *exoA* nodules	334	62	16%	32%
	exo2	up-regulation exclusively in *exoA* nodules	153	10	7%	5%
minor categories						
2.9	N4	maximal expression in 4 dpi nodules and expression in *exoA* nodules	10	0	0%	0%
2.10	fix+_NN	maximal expression in mature nodules, not affected by ammonium nitrate-addition	14	1	1%	1%
2.11	wt nodules	expression only in wild-type *Rhizobium*-induced nodules	3	0	0%	0%
unclear		unclear pattern	179	13	9%	7%

**Figure 4:**
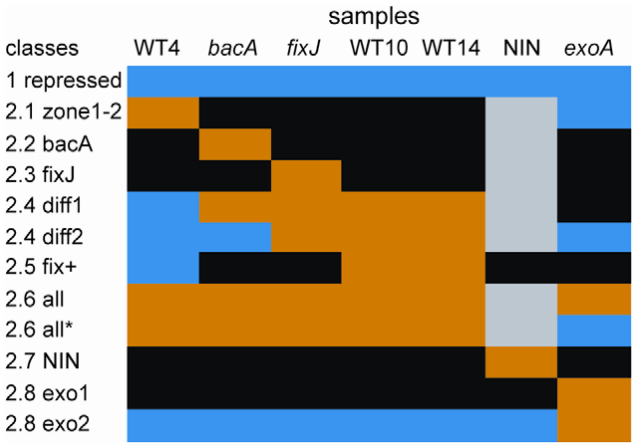
Graphical representation of the major expression classes identified in *Medicago truncatula* nodules. The classes were defined from the genes scored as differentially regulated in *Medicago truncatula* nodules as compared to nitrogen-starved uninoculated roots. Color code: blue = down-regulated in nodules; black = weak induction; brown = maximal induction; grey = not considered for pattern definition.

We distinguished two major classes of repressed (1) and up-regulated genes (2); the latter was sub-divided into different groups according to gene expression patterns at different times and/or in defective nodules, as summarized in Table 2 and Figure 4, and described in more detail as follows.

(1) 1387 gene probes were found to be down-regulated in nodules (“repressed” class). They include the *MtBGLU1* repressed marker gene [27], as well as a series of genes potentially involved in stress responses (e.g. from the phenylpropanoid/flavonoid/phytoalexin pathways), as recently reported [28]. This class notably contains members of a variety of multigene families (e.g. encoding putative transporters, cell wall proteins, enzymes, transcription factors, cytoskeleton proteins, signalling proteins, disease resistance proteins…), for which other members were found to be induced in nodules (see examples in Table S3). This clearly supports the notion of nodule-preferential paralogous genes that was previously suggested from a more limited data set [18].

Eight main classes (2.1 to 2.8) were identified among up-regulated genes, in addition to the minor 2.9 to 2.11 classes (Table 2 and Figure 4).

(2.1) Genes of this first class (also termed “zone 1–2”, 110 probes) were found maximally expressed in 4 dpi immature nodules and were not expressed in *exoA* nodules. Their expression is thus predicted to require the presence of a persistent meristematic zone 1 and/or an infection zone 2. Indeed zones 1 and 2 are smaller than zone 3 in mature nodules and absent from *exoA* nodules at 10 dpi. This conclusion is supported by the previously determined transcript localisation of nine of these genes (*MtENOD11*, *MtENOD40*, *MtN1*, *MtN6*, *MtAnn1*, *EFD*, *MtRR4*, *DAS12*) using either reporter gene fusions or *in situ* hybridization analyses [24], [26], [35]–[41]. The “zone 1–2” genes may be involved in various processes, such as meristem activity, NF signalling, *Rhizobium* infection or cell differentiation. This class includes two genes encoding LOG-like proteins, potentially involved in cytokinin biosynthesis [42], [43] (MT005792 and MT010556; see Table S1 for correspondences with MtGEA affymetrix IDs) and a gene encoding a zeatin-O-glycosyltranferase (MT009525, termed *MtZOG1)*, thought to convert active cytokinins into inactive forms [44]. This is interesting in view of the documented role of cytokinin in nodule initiation and strongly suggests that the levels of active cytokinins are actively controlled in the nodule apex.

The remaining classes were all found to be maximally expressed in 10 dpi or older nodules:

(2.2) The genes of this class (also termed “bacA”, 87 gene probes) were maximally expressed in *bacA* nodules, indicating that bacteroid differentiation is not required for their activation. This group comprised two categories of genes. The first one corresponds to genes that were also well expressed in 4 dpi WT nodules, with some of them previously shown be transcribed in the apical nodule region. We hypothesise that their transcripts accumulate in *bacA* nodules because the nitrogen-fixing zone 3 does not differentiate, thus leading to an enlargement of the zone 1–2. We believe this is the reason why the *MtHAP2.1* [24], *MtENOD12* [45] and *MtMMPL1* [37] nodulin genes were found in this class. This was also the case for the genes encoding DAS18, DAS25 and DNF1 signal peptidases, which are required for targeting NCR/CCP nodule cysteine-rich proteins [46], [47] to bacteroids and essential for symbiosome development [40]. Thirteen NCR genes were found to be transcriptionally activated prior to the onset of bacteroid differentiation (2 genes from class 2.1, 2 from 2.2 and 9 from 2.6). Considering the importance of NCR genes in bacteroid differentiation [48], these NCR genes are very good candidates for triggering the initial steps of this process. One of these (*NCR247*, class 2.1) has been shown to possess antimicrobial activity [48].

**Figure 5:**
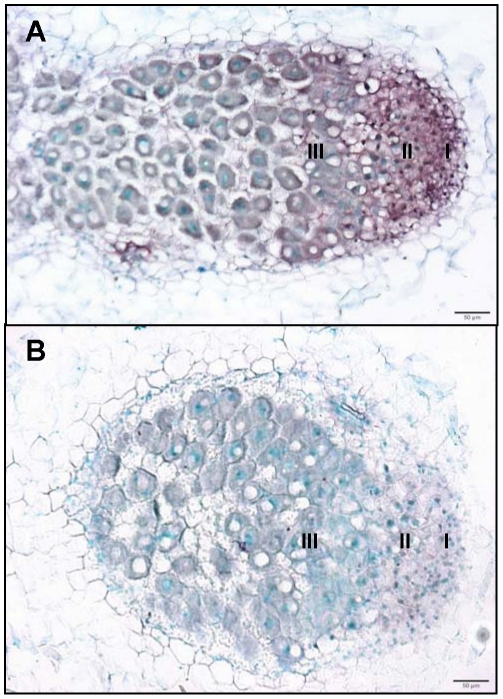
Localisation by *in situ* hybridisation of *MtCKX1* cytokinin oxidase transcripts in *Medicago truncatula* nodule. Use of antisense (A) or sense (B) digoxygenin-labelled RNA probes: hybridization signals were detected only with the antisense probe, in the zone 2 and upper zone 3, and appeared brownish on these methyl green-stained nodule sections. (I), (II) and (III) indicate the nodule zone 1, 2 and 3 respectively. Bar = 50 µm.

**Figure 6:**
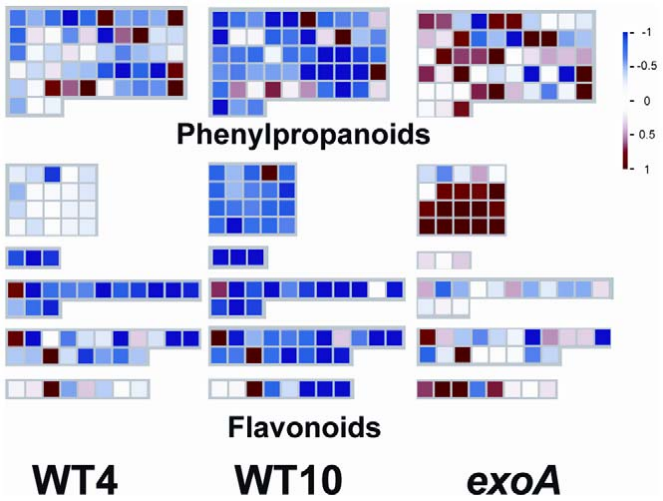
Expression pattern of secondary metabolism genes in wild type and *S. meliloti exoA-*induced nodules. Nodules were harvested at 4 (WT4) or 10 (WT10, *exoA*) days post inoculation and gene expression was analysed using Mt16KPlus microarrays, by comparison to nitrogen-starved uninoculated roots. Each square represents a different gene probe (MapMan representation).

The second category of “bacA” genes was poorly expressed in WT nodules and, similarly to the (2.3) class (termed “fixJ”, 124 gene probes), maximally expressed in *bacA* as well as *fixJ* nodules. Many of these genes (e.g. encoding chitinases, ripening protein-like proteins and cysteine proteases) are likely to be associated with senescence and consistent with previous studies of nodule senescence [22], [49]–[51]. Some of these genes were also expressed in *exoA* or nitrate-treated (NN) nodules (i.e. situations of accelerated senescence), or to a lower extent in WT14 nodules, probably reflecting the initiation of senescence.

(2.4) The largest class of up-regulated genes (termed “diff”, 535 probes) was maximally expressed in *fixJ* and mature WT nodules, but not or weakly expressed in WT4 nodules. We therefore believe that these genes are likely to be associated with the differentiation process giving rise to the nitrogen-fixation zone 3 (not yet present at 4 dpi). A subset of 347 genes (“diff1” subgroup) was already activated in *bacA* nodules, in contrast to the remaining 188 genes (“diff2” subgroup). This suggest that the “diff2” genes, that include the *MtN31* (*NCR158*) and *MtCAM1* marker genes [27], are associated with a later stage of plant cell differentiation accompanying bacteroid differentiation. The NCR/CCP gene family represented a large part of the 2.4 class, with 78 and 64 genes respectively in the diff1 and diff2 subgroups, representing altogether ∼85% of the 168 NCR genes detected on the 16K Plus arrays (Table S1). In addition to the NCR/CCP gene family, the inhibition of the cytokinin signalling pathway via the type A response regulator MtRR4 has recently been proposed to contribute to the triggering of plant and/or bacterial cell differentiation [26]. We found here that a gene putatively encoding a cytokinin oxidase (*MtCKX1*, MT008230), which catalyses the irreversible degradation of cytokinins [43], belongs to the “diff1” group. We then showed by *in situ* hybridisation that *MtCKX1* transcripts are localised between the apical nodule zone 2 and the beginning of zone 3 (Figure 5). This observation is consistent with the hypothesis that differentiation of tissues underlying the nodule meristem requires decreasing cytokinin activity [26]. This could be achieved by complementary pathways involving three genes expressed in the nodule zone 2: *MtRR4* (inhibition of the signalling pathway), *MtZOG1* (cytokinin conjugation) and *MtCKX1* (cytokinin degradation).

(2.5) Genes of the “fix+” group (159 probes) were clearly associated with the nitrogen fixation process, since they were only expressed in mature wild-type nodules and were down-regulated by NH_4_NO_3_ addition. As anticipated, this class was found to comprise genes involved in the nitrogen assimilation pathway (e.g. glutamine synthase, glutamate synthase, asparagine synthase, aspartate aminotransferase) [52]. Surprisingly, we also found that *DMI1* (MT011705), well known for its key role in NF signalling [53], [54], belonged to this class. This raises intriguing questions regarding the possible involvement in late symbiotic stages of genes identified and characterized so far only for their early symbiotic roles. Similar questions are raised by the expression patterns of *MtSYMREM1* and *NIN* (see below).

(2.6) Genes of this class (also termed “all”, 267 gene probes) were expressed in most nodule samples. It is likely that some of these genes are expressed in tissues for which the relative abundance remains similar irrespective of the nodule developmental stage or age. Thus *ENOD2* is expressed in the nodule parenchyma at the basis and periphery of the nodule [55], while *MtTubb1*, a β-tubulin gene, has been shown by promoter:GUS fusion analysis to be expressed in most nodules tissues [50]. Some genes may also be expressed in different nodule tissues, like the remorin gene *MtSYMREM1*, found to be associated both with the infection process and with symbiosomes [56], or *NIN*, shown by *in situ* hybridization to be expressed in a region extending from zone 1 to the distal region of zone 3 in pea nodules [57]. A rather large number of genes (172) were also expressed in empty *exoA* nodules at 10 dpi, indicating that they can be expressed independently of bacterial infection. This could be anticipated for genes contributing to nodule organogenesis such as *MtNIN*, but was unexpected for genes like *MtSYMREM1*, the lectin gene *MtLEC4* or a small subset of six NCR genes (see Table S1). Different roles may therefore have to be envisaged for such genes or gene families.

(2.7) Genes of the “NN” (for Nitrate-treated Nodules) class (75 probes) were maximally expressed following NH_4_NO_3_ addition and comprised genes potentially involved in NH_4_NO_3_ assimilation (e.g. two glutamate dehydrogenase genes: MT000848 and MT015442) or remobilisation processes (e.g. a beta amylase and five heat-shock protein genes). This group also included a non symbiotic haemoglobin-like gene (MT000284), in contrast to the down-regulation of all the other leghemoglobin genes (Table S1 and Figure 2). This is a good example of mutually exclusive expression patterns within gene families. A few genes (termed “fix+_NN” in Table 2) were well expressed both in N-fixing nodules and NN nodules, such as two asparagine synthase genes, and may therefore be involved in the assimilation or transport of nitrogen, whether resulting from symbiotic fixation or nitrogen uptake.

(2.8) This last class corresponds to genes maximally (“exo1” subgroup, 334 genes) or exclusively (“exo2” subgroup, 153 genes) expressed in *exoA* nodules. Many of these genes are involved in secondary metabolism, and notably in flavonoid and phenylpropanoid pathways (see a MapMan representation in Figure 6). This class includes 14 out of the 20 genes most strongly induced by an elicitor (invertase) in *M. truncatula* suspension cells [58]. Substantial differences between *exoA* and WT nodules were also found amongst the so-called “biotic stress” genes (MapMan classification). These include genes for a variety of proteins involved in resistance to pathogens: NBS-LRR proteins [59] (with 24 differentially affected genes), RAR1 (MT003202), an important component of the resistance specified by NBS-LRR proteins [60], homologues of the EDS1 (MT007863) and PAD4 proteins (MT007233) which function together in plant immunity [61], disease resistance response proteins, polygalacturonase inhibitor proteins (five probes), and a NADPH oxidase (MT014380). This class also comprises two genes playing a key role in jasmonic acid biosynthesis [62] encoding allene oxide synthase and cyclase respectively (MT005924 and MT000081). These observations support and extend previous studies suggesting an important role for *Rhizobium* surface polysaccharides in limiting plant defence reactions [28], [63]–[65]. However, the activation, albeit at a lower level, of a substantial fraction of defence-like genes in WT nodules (“exo1” subgroup) raises interesting questions regarding their role during normal nodule development, discussed below in the section on regulator genes.

The most recent transcriptomic analysis of the nodulation programme in *M. truncatula* [28] has identified five activation profiles from a time course study of nodule development, with symbiotic mutants being analysed independently. Here we have combined data for both normal and defective nodulation, which has led us to define eight major activation patterns. A comparison of these two studies reveals the following information.

Our 2.1 (“zone 1–2”) class corresponds essentially to profiles 4 and 5 described in [28], i.e. genes maximally expressed in immature nodules. The remaining three profiles (profiles 6 to 8) were described in [28] as representing genes expressed in mature nodules. We examined a set of 100 up-regulated genes from profiles 6–8 shared in both studies (using the LEGoo portal to find the correspondence between MtGI7 TC [28] and Mt16KPlus oligonucleotide identifiers). We found that there was an overall good consistency between both studies, with Profile 6 genes found in many cases in earlier expression classes (2.1 and 2.2) than Profile 7 and 8 genes (mostly found in 2.4). However the fact that we integrated data from defective nodules allowed to distinguish more expression patterns than in [28].

It has been proposed in [28] that nodulation essentially involves two phases of gene expression reprogramming. The results from our study allowed us to further identify four major phases of activation/reprogramming related to: (i) the early nodule symbiotic zones 1 and 2 (class 2.1 and part of 2.2) and potentially involved in early NF signalling and/or bacterial infection; nodule zone 3 differentiation, either (ii) preceding (“diff1” genes) or (iii) dependent upon bacteroid differentiation (“diff2” genes); (iv) nitrogen fixation (“fix+” genes). In addition to these four specific phases, we identified a fifth set of genes expressed either during several stages or in several tissues (class 2.6 “All”), as well as two subsets associated with senescence (part of class 2.2 “bacA”, 2.3 “fixJ”, 2.7 “NN” and 2.8 “exo”) or plant stress/defence responses (Class 2.8).

### Identification and specificity of regulatory genes associated with the different nodule expression patterns

Having identified distinct patterns of gene expression in nodules, we were interested in the associated regulators of gene expression, and notably TFs. We identified 192 genes up-regulated in nodules (Table S4) that belong to 35 gene families encoding putative TFs and proteins involved in post-transcriptional regulation, chromatin remodelling or protein degradation (Table 3). Seven TF families were highly represented, with between 9 and 24 gene probes each. The representation of five of these (Myb, bHLH, C2H2, NAM/NAC, HB = homeobox families) correlated with the known size of the gene family in *M. truncatula* [4], whereas members of the ERF and WRKY families were several-fold over-represented.

**Table 3:** Thirty-five classes of gene expression regulators detected as up-regulated in *Medicago truncatula* nodules.

protein family	regulated process	induced genes	regulation classes	major regulation classes
basic helix-loop-helix (bHLH)	transcription	13	6	2.8: exo1 (6)
bZIP	transcription	7	4	2.4: diff1/2 (4)
CCAAT-binding TF	transcription	5	4	
CCR4-NOT	transcription	3	3	2.4: diff1/2 (2)
DNA-binding protein-like	transcription	3	3	
ERF/AP2	transcription	24	7	2.6: all (9), 2.5: exo1/2 (9)
G2-like	transcription	1	1	
GRAS	transcription	6	3	2.8: exo1 (4)
HB	transcription	9	5	2.4: diff (5)
HSF	transcription	2	2	
KH	post-transcriptional	1	1	
LIM domain-containing protein	transcription	1	1	
LOB domain protein	transcription	1	1	
MADS box	transcription	1	1	
TFIIS	transcription	1	1	
Myb	transcription	18	9	2.4: diff1/2 (4), 2.8: exo1/2 (4)
no apical meristem (NAM)	transcription	12	6	2.4: diff1/2 (4)
ovate protein family	transcription	1	1	
RWP-RK domain protein	transcription	1	1	
S1FA	transcription	1	1	
SAR DNA-binding protein	transcription	1	1	
TFIIS containing protein	transcription	1	1	
WRKY	transcription	24	1	2.8: exo1/2 (20)
Zinc finger (Ran-binding)	nuclear protein import	1	1	
Zinc finger, AN1-like	protein degradation	4	4	
Zinc finger, B-box type	transcription	3	2	
Zinc finger, C2H2 type	transcription	10	5	2.8: exo1 (4)
Zinc finger, CCCH-type	post-transcriptional reg.	7	6	
Zinc finger, CCHC-type	unclear	1	1	
Zinc finger, DHHC type	unclear	1	1	
Zinc finger, Dof-type	transcription	1	1	
Zinc finger, FYVE/PHD-type	unclear	1	1	
Zinc finger, GRF-type	unclear	1	1	
Zinc finger, RING-type	protein degradation	23	6	none
Zinc finger, TAZ-type	chromatin remodeling	2	2	

The number of induced genes is indicated as well as the number of corresponding regulation classes. Figures in parentheses indicate the number of genes.

We examined the distribution of these 192 regulatory genes within the expression classes that we have defined (Table 2, 3 and S1) as well as other expression data available in the MtGEA (V2, July 2009 release; 64 experiments, 156 Affymetrix gene chips) and LEGoo databases (October 2009 release; data from 42 publications on *M. truncatula*, based upon various transcriptomic tools including three generations of microarrays). Data mining revealed that only six regulators (MT000013, MT003698, MT006389, MT007392, MT009598, MT015410) were also up-regulated during the arbuscular mycorrhizal symbiosis [29], [66], [67]. These were distributed between six TF families and four regulation classes (“bacA”, “diff2”, “fix+” and “NN”) and all of them were also expressed in non symbiotic conditions.

A small number of regulatory genes were predicted to be expressed preferentially in the nodule apical region, with five “zone 1–2” genes and six “bacA” genes including *MtHAP2.1* [24]. Four of these were found to be nodule-specific (based on current MtGEA data) or nodule-preferential (Table 4). Two new CCATT-binding factors were identified, with one “zone 1–2” *MtHAP5* (MT007765) also expressed in other organs (notably seeds), and one other “bacA” *MtHAP2* (termed *MtHAP2.2*, MT016112) expressed at a lower level in nodules and a much higher level in roots compared with *MtHAP2.1*. By looking for co-expressed genes using the MtGEA data (Pearson correlation coefficient ≥0.8) we identified three additional candidate regulatory genes with a similar expression pattern (Table 4 and Table S5), although none of these are nodule-specific. One of these genes encodes a WRKY TF, described to be down-regulated by the pathogenic signal methyl jasmonate [68], [69].

**Table 4:** Regulators of gene expression mostly or preferentially detected in *Medicago truncatula* nodules.

16KPLus ID	affy ID	source	name, regulator family	class	maximum induction ratio (arrays)	maximum induction ratio (Affy)
MT010767*	Mtr.14503.1.S1_at*	16KPLus	MtN20, Zinc finger, GRF type	zone1-2	7.4 (N4)	644 (N4)
MT009625	Mtr.38198.1.S1_at	16KPLus	Zinc finger, RING-type	zone1-2	1.6 (N4)	1.7 (N4)
MT003118	Mtr.41581.1.S1_at	16KPLus	EFD, ERF/AP2	zone1-2	4.4 (N4)	54.3 (N4)
MT003118	Mtr.27549.1.S1_at	MtGEA	WRKY	zone1-2 like		16 (N4)
	Mtr.39406.1.S1_at	MtGEA	AP2/EREBP	zone1-2 like		25 (N4)
	Mtr.14760.1.S1_at	MtGEA	basic helix-loop-helix (bHLH)	zone1-2 like		41 (N4)
MT008842*	Mtr.43750.1.S1_at*	16KPLus	MtHAP2.1, CCAAT-binding transcription factor	bacA	4.5 (N4)	453 (N4)
MT013353	Mtr.19842.1.S1_at	16KPLus	GRAS	fixJ	1.9 (fixJ)	4.7 (NN)
MT002933*	Mtr.38404.1.S1_at*	16KPLus	Zinc finger, C2H2 type	diff1	4.0 (N10)	170 (N10)
MT002627	Mtr.43742.1.S1_at	16KPLus	bZIP	diff1	2.9 (bacA)	4.2 (N10)
MT007902	Mtr.19536.1.S1_at	16KPLus	no apical meristem (NAM)	diff1	2.5 (N10)	4.6 (N10)
MT016328	Mtr.41968.1.S1_at	16KPLus	no apical meristem (NAM)	diff1	2.1 (N10)	32 (N10)
MT014036	Mtr.37567.1.S1_at	16KPLus	Homedomain protein	diff1	2.0 (N10)	5.6 (N10)
	Mtr.23988.1.S1_at	MtGEA	basic helix-loop-helix (bHLH)	diff-like		37 (N10)
	Mtr.9532.1.S1_at	MtGEA	no apical meristem (NAM)	diff-like		8.1 (N10)
	Mtr.31954.1.S1_at*	MtGEA	GRAS	diff-like		25 (N10)
	Mtr.31955.1.S1_at*	MtGEA	GRAS	diff-like		36 (N10)
MT016186*	Mtr.28107.1.S1_at*	16KPLus	cAMP response element binding prot.	fix+ or fix+_NN	1.6 (N10)	3.5 (N10)
MT007392	Mtr.43099.1.S1_at	16KPLus	Myb	fix+	1.7 (N14)	1.8 (N10)
MT011890	Mtr.9880.1.S1_at	16KPLus	Zinc finger, DHHC type	fix+	1.6 (N10)	4.7 (N10)
	Mtr.20585.1.S1_at	MtGEA	Homeodomain protein	fix+-like		2.3 (N14)
MT010803*	Mtr.7232.1.S1_at*	16KPLus	MtC00553, unclear	all	15.6 (bacA)	236 (N14)
MT016467*	Mtr.28094.1.S1_at*	16KPLus	MtNIN, RWP-RK domain-containing protein	all	4.0 (bacA)	98 (N14)
MT001831	Mtr.43700.1.S1_at	16KPLus	Homeodomain protein	all	2.8 (N10)	13.2 (N10)
MT002843	Mtr.9513.1.S1_at	16KPLus	Myb	all	2.1 (N10)	10.8 (N10)
MT012710	Mtr.10993.1.S1_at	16KPLus	basic helix-loop-helix (bHLH)	all	1.9 (fixJ)	3.7 (N4)
MT001831	Mtr.43700.1.S1_at	16KPLus	Homeodomain protein	all	2.8 (N10)	13.2 (N10)
MT009243	Mtr.10987.1.S1_at	16KPLus	ERF/AP2	all	2.2 (bacA)	2.8 (N14)
	Mtr.18387.1.S1_at	MtGEA	Zinc finger, C2H2 type	all-like		117 (N10)
MT010468	Mtr.985.1.S1_at	16KPLus	ERF/AP2	unclear	1.5 (N10)	2.9 (N4)
MT015410	Mtr.12441.1.S1_at	16KPLus	basic helix-loop-helix (bHLH)	NN	1.5 (NN)	2.2 (NN)
MT016119	Mtr.44189.1.S1_at	16KPLus	Homeodomain protein	unclear	1.7 (N10)	2.6 (N10)

Are indicated the observed ratios between the level of expression in nodules and nitrogen-starved non-inoculated roots, as determined by Affymetrix chips or 16KPlus microarrays. The gene probes identified from the *M. truncatula* gene atlas were found by searching genes that were co-expressed with nodulation-specific gene probes (as described in Table S5). The expression pattern of these genes was classified using the gene atlas data i.e. without mutant nodules. Parentheses in the “ratio” columns indicate the condition of maximum expression. Asterisks indicate genes the expression of which appeared to be nodule-“specific” or highly preferential.

The “diff” class included significantly more candidate regulatory genes (39 genes belonging to 16 regulator families), encoding notably five homeodomain proteins and a member of the plant-specific ovate protein family, described to interact with homeodomain proteins [70], which is also strongly expressed in other organs. Interestingly, a third *HAP2* gene (*MtHAP2.3*; MT005075), maximally expressed in seeds, was found in the “diff1” subgroup. Two genes that are highly nodule-preferential, encoding a zinc finger C2H2-type protein (MT002933) and a NAM TF (MT016328) (Table 4), are likely to be of particular interest for studying nodule differentiation due to their putatively specialized functions in this process. Using the MtGEA data we were also able to identify two new GRAS TFs that are co-expressed with the “diff” genes and also up-regulated (six and nine-fold respectively) during the endomycorrhizal symbiosis (Mtr.31954.1.S1_at, Mtr.31955.1.S1_at, Table 4).

Amongst the 12 identified “fix+” regulators, only one (MT016186), although weakly expressed, seems to have a nodulation-specific expression pattern while two are nodule-preferential (Table 4), but with a modest activation ratio. This suggests that the regulators accompanying the symbiotic N-fixation process are for a large part shared with other metabolic pathways or plant processes. Similarly, the addition of ammonium nitrate to mature nodules (“NN” genes) did not lead to activation of nodule-specific regulators. The presence of two MYB TFs (MT005520, MT002428), previously reported to be up-regulated by N satiety in *M. truncatula* split-roots [71], suggest a direct link with the ammonium nitrate treatment. These TFs may also contribute to regulating the remobilisation processes accompanying the arrest of nitrogen fixation and the redirection of metabolites from nodules to the rest of the plant.

The “all” class (32 regulator genes from 15 families) comprised two nodulation-specific regulators (Table 4) including *MtNIN*, well known to play an essential role in nodule initiation [57], [72], [73]. This class also contained a second *MtHAP5* gene (MT000406), expressed in many other organs (shoots, flowers, seeds…) and conditions. It thus appears that several *MtHAP2* and *MtHAP5* genes have different expression patterns in nodules, potentially leading to various combinations of oligomeric CCATT-binding complexes. It will now be interesting to investigate whether such complexes may have different roles at different stages of nodule development (or in different tissues).

This global survey of nodule-associated regulators thus allowed us to identify 32 genes specifically or maximally expressed during nodulation, including nine genes indirectly identified from the MtGEA. Thus only a small percentage of the regulator genes found by 16KPlus microarrays were either nodule-“specific” or nodule-preferential, suggesting that most nodule-associated regulators have been recruited from other genetic programs. Many nodule regulators are also expressed in either roots or seeds, as well as in response to plant pathogens or pathogenic signals and in response to salt stress (22% and 18% shared regulators respectively, based on LEGoo data, Table S6). However, the “zone1-2”, the “diff” and the “fix+” classes shared very few regulators with either pathogen or salt stress responses (2 out of 57 regulator genes in total), in contrast to the “exo” class (approximately 65% of shared regulators). This is consistent with the fact that the “zone1-2” and “diff” genes are much more nodulation specific.

The “exo” class contained the largest number of candidate regulator genes (a total of 72 from 19 regulator families), but none were nodulation-specific. These included most of the nodule-associated WRKYs (21 out of 24 probes), a family of TFs often involved in stress responses [74]. LEGoo interrogation revealed that 44% and 30% of the “exo1” and “exo2” regulators respectively were up-regulated in response to pathogens/pathogenic signals (see Table S6 and Figure S1 for an example). It has been shown that different subsets of *M. truncatula* TFs are induced following treatment either with yeast elicitor (a pathogen mimic) or methyl jasmonate (a wound/necrotrophic pathogen signal) [68]. We found that both subsets of TF were induced in the “exo1” class, with seven members from five TF families and notably the most strongly activated TFs for each subset (a WRKY TF: MT006670 and a bHLH TF: MT009684, induced respectively 600-fold by yeast elicitor and 240-fold by methyl jasmonate [68]). We also found a possible ortholog of the best characterized TF in jasmonate signalling, AtMYC2 (MT009684 bHLH), also reported in a previous study to be induced during nodulation [68]. Several other typical markers of the jasmonate pathway were activated in both WT and defective nodules: a jasmonate ZIM-domain (JAZ-like) protein (MT002288) and four of the TFs most strongly induced by jasmonate (one HD-ZIP: MT008226; two WRKY: MT015782, MT014097; one AP2/ERF: MT004784) (Table S6).

Jasmonates are a family of oxylipins that regulate plant responses to environmental and developmental cues [75], [76] and for which positive and negative effects have been reported in relation to root endosymbioses [77]. Jasmonate has been shown to inhibit the NF signalling pathway in *M. truncatula* roots [78] and proposed on the basis of indirect evidence (induction of lipoxygenase genes) to be involved in nodule senescence [22]. The fact that jasmonate appears to be produced in normal nodules, even at an immature stage (4 dpi) suggests that this plant hormone may have a wider role than suspected so far. Jasmonate has been proposed to mediate salt stress responses in the *M. truncatula* root apex [79], which may explain, at least in part, why numerous salt-induced genes and regulators are common to root nodulation. Characterizing mutants affected in the production or perception/signalling of jasmonate would be very useful to clarify its role(s) during nodulation, which may of course differ depending upon the developmental stage and tissue.

While it is well established that there is an inhibition of defence reactions in the root during initial interactions with *Rhizobium*, our data raise the question of a possible (re)activation of defence or stress responses during nodule development. This is more pronounced in non-infected *exoA* nodules and it can be considered that “exo2” genes which are activated in only these aberrant nodules are not informative about WT nodules. However “exo1” genes are also expressed in WT nodules, including at 4 dpi, i.e a stage when nodules are actively developing. Reactivation of gene expression for several defence genes at 72 hpi, following an initial down-regulation during the first 48 h, has already been reported [17], and includes one WRKY gene (BG582680) that belongs to the “exo1” group (MT01497). However, we cannot exclude the possibility that the nodule harvesting process may be sufficient in itself to induce stress-associated genes (see e.g. [80]). Considering the homology between NF receptors and receptors involved in defence-related perception of chitin oligomers [81], or between NCR peptides and antimicrobial cysteine-rich peptides [48], it is also conceivable that certain “exo1” regulators have been recruited from defence pathways and contribute either to nodule development or the control of rhizobia.

### Identification of different patterns of symbiotic gene activation in roots

Following the identification of different gene expression patterns and regulators in nodules, we decided to further analyse the expression of certain regulator and nodulation marker genes during early symbiotic stages in roots. For this, we carried out quantitative RT-PCR, a highly sensitive and inexpensive method convenient for analysing multiple samples.

To analyse gene expression during early pre-infection stages, we used NF-treated roots (10^−8^ M NF, 24 h treatment) of the supernodulating double *sunn-skl* mutant, previously shown to be significantly more reactive to NFs as compared to WT *M. truncatula* [82]. To study gene expression during the symbiotic association with *Rhizobium*, we analysed WT *M. truncatula* roots harvested at 0, 1, 2 and 3 dpi. Expression analyses (1 and 3 dpi with *S. meliloti*) in symbiotic mutant backgrounds were also included in order to define groups of genes involved in early pre-infection or later infection/nodule organogenesis. The *nfp* mutation (*nfp-1* and *nfp-2* alleles), defective in all NF-dependent processes [83], [84] including symbiotic gene activation [16] was used as a negative control. The symbiotic plant or bacterial mutants *hcl-1*, *lin* and *exoA* affected in downstream infection and nodule organogenesis processes were also included. *hcl-1* (a mutant allele of the putative NF receptor LYK3), *lin* (lumpy infection mutant, defective in a host U-box protein, [85], [86]) and *S. meliloti exoA* mutants all display defective infection phenotypes. *exoA* and *hcl-1* are blocked at the stage of infection thread initiation while infection thread progression in root hairs is limited in the *lin* mutant. In these three mutants early NF signalling and cortical cell activation are not seriously modified [32], [86]–[88]. Nodule primordia are initiated normally in *lin* and in response to *exoA* inoculation, but further nodule development is impaired in *lin*, while non-infected *exoA* nodules stop growing after several days. Finally, we used *efd-1*, a deletion mutant of the AP2/ERF transcription factor EFD, in which infection and nodule formation are initiated normally, but where impaired negative feedback regulation leads to higher number of nodules then for WT plants [26].

We obtained data for a total of 78 genes encoding known nodulation markers, gene expression regulators and signalling genes (Table S7). Most of these genes were taken from the 16KPlus data, including members from eleven of the regulation (sub)groups that we defined earlier. We also included six genes previously identified with 16 K microarrays as NF-induced [82] and two genes selected from a set of cDNA sequences generated from NF-treated purified root hairs (F. de Carvalho-Niebel and A. Niebel, unpublished data). We first validated our system by showing that the expression of nine genes in response to NF or *S. meliloti* (activation for *MtAnn1*, *MtENOD11*, *MtENOD40*, *MtHAP2.1*, *MtNIN*, *MtNIP1*, *MtN24* and *MtTUBB1* and repression for *MtBGLU1*) was similar to previously published data [16], [19], [38], [88]–[90].

**Figure 7:**
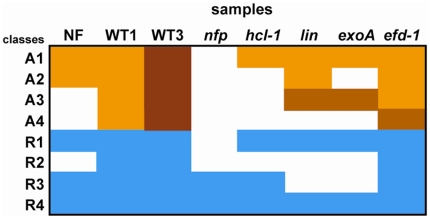
Graphical representation of the different expression patterns identified in *Medicago truncatula* roots. Classes were defined from the genes scored as differentially regulated in *Medicago truncatula* roots by Q-RT-PCR analyses, following 10^−8^ M NF addition (24 h incubation, in the *sunn sickle* double mutant background) or *S. meliloti* inoculation as compared to control roots. WT1 and WT3 respectively mean one or three day post-inoculation with wild type *S. meliloti*. Color code: blue = down-regulated; white = no differential expression; brownish = up-regulated (the darkest corresponding to maximal induction).

The seven tested genes from the “repressed” nodule class were either repressed or non-differentially regulated in roots. Two repression patterns were observed amongst these genes (Table 5, Table S6, Figure 7). The first one, termed R1, has already been described for *MtBGLU1* [16], for which repression is triggered by purified NF and dependent on a functional NF signalling pathway since abolished in the *nfp* mutant. The second one, termed R2, was exhibited by *LYK3*, the expression of which was repressed by inoculation with *S. meliloti* but not by treatment with purified NF. This repression was not observed with *exoA*, *hcl-1*, and *lin* mutants, strongly suggesting that it is directly linked to the *Rhizobium* infection process. From a biological point of view, turning down the expression of the NF entry receptor by *S. meliloti* infections may contribute to controlling new rounds of infections by *Rhizobium* and may thus participate in the negative feed back inhibition of infections and nodulation.

**Table 5:** Definition of different activation patterns in *Medicago truncatula* roots.

	Pattern	NF	WT 1 dpi	WT 3 dpi	*nfp*	*hcl-1*	*lin*	*exoA*	*efd-1*
activated	A1	+	+	+/++	0	+	+	+	+
activated	A2	+	+	++	0	0	+	0	+
activated	A3	0	+	++	0	0	+−	+−	+
activated	A4	0	+	++	0	0	0	0	+/+−
repressed	R1	−	−	−	0	−	−	−	−
repressed	R2	0	−	−	0	0	0	0	−
repressed	R3	−	−	−	−	−	0	0	−
repressed	R4	−	−	−	−	−	−	−	−

+, − and 0 respectively mean induction, repression and no differential expression as compared to control roots. +− means a weaker induction than in the wild type (WT) situations, while ++ indicates the highest level of expression. +/++ indicates that some genes from this class had a + level of expression while others had a ++ level; similarly +/+− indicates that some genes had a + level while others had a +− level. NF stands for response to 10^−8^ M purified Nod factors (24 h treatment), WT for response to WT *S. meliloti*. *nfp*, *hcl-1*, *lin*, *exoA* and *efd-1* indicate the tested plant or bacterial mutants.

Only a fraction of the genes up-regulated in nodule were found to be also activated in roots. Many of the genes predicted to be expressed in nodule zone 1 or 2 were induced in roots, with 9/9 tested “zone1-2” (2.1) genes and 6/13 “all” (2.6) genes. By contrast, only a small proportion (5/46) of the genes belonging to the other tested regulation classes (2.2, 2.4, 2.5, 2.7, 2.8) were found to be up-regulated in roots (Table S7). This is consistent with the fact that the processes related to symbiotic signalling and infection occur both in the root and in the nodule zone 1 and/or 2, in contrast to processes involved in late nodule development or activity. A few exceptions were observed, with notably the leghemoglobin gene *MtLb1* (“diff2” class), induced very early by NFs albeit at a level about 1000 fold lower as compared to mature nodules. Early leghemoglobin gene induction has already been described in *Vicia sativa* and hypothesised to be linked to increased respiration of NF-treated root hairs [91].

Four activation patterns could be distinguished in roots (Table 5 and 6, Figure 7), thus defining distinct early developmental stages. The first two correspond to genes induced by purified NF and whose activation is dependent on a functional *NFP* gene. Pattern A1 genes are *HCL (LYK3)*-independent and therefore involved in pre-infection. They include several early nodulin genes shown before to be expressed during the preinfection stage or induced by NF, like *MtENOD11* [36], *MtAnn1* [38], *MtENOD40* [39], *MtNIN* [73] and *MtHAP2.1* [82]. This group also comprised two MYB TF-encoding genes, one previously found to be NF-induced [82] and a novel gene from the NF-treated root hair cDNA library. Other interesting members of this group are several genes encoding membrane-associated proteins, including *MtN21* and *MtN24* (two nodulation specific genes, first described in [90]) and a new receptor-like kinase gene identified from the root hair library (highly homologous to *AtRLK1*). These proteins may play a role at the plasma membrane level, possibly in relation to the NF perception/signalling machinery or phytohormone transport.

**Table 6:** Expression patterns in roots of nodule- or root hair-associated genes.

nodule regulation class	Identifier	origin	annotation	root pattern
zone1-2	MT008495	16K+	MtAnn1 annexin	A1
zone1-2	MT007328	16K+	MtENOD40	A1
zone1-2	MT016468	16K+	MtENOD11	A1
zone1-2	MT008842	16K+	MtHAP2.1; nodulation-specific	A1
zone1-2	MT016469	16K+	MtN2 (MATH, TRAF domains); nodulation-specific	A1
zone1-2	MT001725	16K+	MtN1-like, nodulation-specific	A2
zone1-2	MT010767	16K+	MtN20 (Zinc finger, GRF type); nodulation-specific	A3
zone1-2	MT006735	16K+	Myb	A3
zone1-2	MT010452	16K+	MtN19; nodulation-specific	A4
zone1-2	MT003118	16K+	EFD (ERF)	A4
all	MT016467	16K+	MtNIN	A1
all	MT008693	16K+	MtN24 (membrane protein); nodulation-specific	A1
all*	MT007813	16K+	MtTubb1 tubulin	A1
all*	MT002234	16K-NF	MtN21 (transporter-like family); nodulation-specific	A1
all	MT000633	16K+	GH3 auxin response gene	A2
all	MT007335	16K+	MtLEC4	A4
all*	MT003955	16K+	bHLH	A4
diff1	MT007526	16K+	MtNip1 (Nodulin 26)	A1
diff2	MT015113	16K+	MtLb1 leghemoglobin; nodulation-specific	A1
exo1	MT009947	16K+	DNA-binding WRKY	A3
nd	MtC70568	rh cDNAs	MYB (LAF1-like)	A1
nd	MtC70235	rh cDNAs	RLK receptor like kinase	A1
nd	MT003798	16K-NF	MYB (SHAQKYF class)	A1

rh cDNA means Nod factor-treated root hair cDNA library; 16K-NF means search of Nod factor-induced genes with 16 K microarrays [82].

Pattern A2 corresponds to genes that were not induced in *hcl-1* and responded poorly to the *exoA* mutant, thus suggesting a role in early steps of infection thread formation. This pattern was exhibited by two genes: a *GH3* auxin-responsive gene and a gene homologous to the infection-associated *MtN1* gene [41] which is strongly induced (19-fold) by nitrogen starvation [71], a condition necessary for successful nodulation. These two genes represent potential markers for the NF entry receptor pathway [88], [92]. The fact that *GH3* is strongly induced by auxin suggests that this stage may be correlated with auxin accumulation in the root. The fact that *GH3* and *MtN1*-like genes were not activated following inoculation with the *S. meliloti exoA* mutant may be due to the inability of this mutant to prevent plant defence reactions.

Other categories of genes were activated later during the interaction since they were not induced following 24 h NF treatment. Their activation was *HCL-*dependant and either detectable or absent in the *lin* mutant (Pattern A3 and A4 respectively). Pattern A4 genes (e.g. *MtLEC4*, *MtN19* and *EFD*) are thus likely to be activated after Pattern A3 genes (e.g. *MtN20*). *MtN19* activation was strongly reduced in the *efd-1* mutant both in roots (this study) and in nodules [26], making it a possible target gene of EFD. The role of *MtN19* might thus be related to negative feedback inhibition of nodule initiation, as proposed for *EFD* in the root.

A set of nodule-induced genes were found to be repressed in *S. meliloti*-inoculated roots. This repression did not rely upon a functional *HCL* gene but was *LIN*-dependent, thus defining a new repression pattern termed R3. Most of these genes were from the “*exo”* class. This LIN-dependent/NF pathway-independent repression was generally not observed with the *exoA* mutant. Data mining with LEGoo showed that a significant proportion of these repressed genes are induced by plant pathogens or pathogenic signals. LIN may therefore be involved in the down-regulation of defence-like genes in roots, required to permit *S. meliloti* infection. This would be entirely consistent with the hypothesized altered regulation of plant defence during rhizobial invasion of *lin* [86]. The absence of bacterial EPS (as in *exoA*) would prevent this down regulation and consequently prevent *S. meliloti* infection. It will now be interesting to evaluate whether LIN is on a (so far uncharacterised) pathway involved in EPS signalling which is critical for successful infections.

Finally, a single gene from the NN class possessed a distinct repression pattern (R4) which was unaffected by any of the tested mutations. This gene encodes a transcription factor up-regulated by N satiety [71] and therefore likely to be positively controlled by a signal linked to the plant N nutritional status. This would explain why its expression decreases in NH_4_NO_3_-starved plants.

In conclusion, by global analyses of gene expression using both WT and symbiotic mutant material, we have identified distinct patterns of gene activation or repression in roots and nodules, complementary to previous studies [17], [27], [28], as well as associated candidate regulators. This and other studies aim to define the temporal order of activation/repression and the hierarchy of multiple gene expression within the complex symbiotic programme. We can anticipate that approaches based on physical dissection and transcriptome analyses of defined root or nodule tissues will also be very useful for the understanding of gene networks associated to nodule development and activity. This work also shows the necessity and value of efficient data mining tools. Those are critical to combine various sources of information, cross-compare genetic programmes involved in different plant responses and developmental pathways, and provide a solid basis for selecting genes for further functional studies.

## Materials and Methodsd>

### Plant growth and Bacterial strains

*M. truncatula* cv Jemalong A17 (wild type, *nfp-1*, *nfp-2*, *hcl-1*, *lin* and *efd-1* lines) was grown in aeroponic caissons as described in [93], with the following chamber conditions: temperature: 22°C; 75% hygrometry; light intensity: 200 µE.m^−2^.s^−1^; light-dark photoperiod: 16 h–8 h. Wild type *S. meliloti* RCR2011 pXLGD4 (GMI6526), *S. meliloti* RCR2011 *exoA* pXLGD4 (GMI3072), *S. meliloti* RCR2011 *bacA* pXLGD4 (GMI11491) and *S. meliloti* RCR2011 *fixJ* pXLGD4 (GMI5730) were grown at 28°C in tryptone yeast medium supplemented with 6 mM calcium chloride and 10 µg.mL^−1^ tetracycline. For microarray analyses, plants were grown for 2 weeks in caisson growth medium [36] supplemented with 10 mM NH_4_NO_3_, then for 3 days in nitrogen-free caisson growth medium before inoculation. For Q-RT-PCR experiments, seedlings were grown for 5 days in nitrogen-free caisson growth medium before inoculation. Caissons (10 L of growth medium) were inoculated with 10 mL of bacterial culture diluted to OD_600_ = 1 in nitrogen-free plant growth medium. For nodule harvesting, segments of nodulated root systems were kept on ice for a few minutes while nodules were cut out; those were immediately frozen in liquid nitrogen and stored at −80°C. Each nodule or control root sample was harvested from 50 plants, each biological repetition corresponding to a different caisson.

### Microarray analyses

Total RNA was extracted with the Trizol RNA extraction kit (Invitrogen) then purified on a Microcon-30 column (Millipore) and RNA quality was checked using a Bioanalyser (Agilent Technologies). Sixteen micrograms of total RNA were used to generate Cy3 and Cy5-labelled cDNAs as in [29]. The Mt16KPlus microarrays are described at http://www.ebi.ac.uk/arrayexpress (accession number A-MEXP-138). Hybridization of targets, image acquisition and analysis were carried out following [29], using an ASP hybridization station (Amersham Biosciences) and the ImaGene 5.5 software (Biodiscovery, Los Angeles). Four repetitions were analysed for each nodule sample, using a same reference sample (nitrogen-starved non inoculated roots) for all microarray experiments. All data is MIAME compliant; the raw data has been deposited in the MIAME compliant ArrayExpress database (accession number: E-TABM-688). Data were processed as indicated in [26] (normalization, t-statistics and multiple-comparison corrections with the Benjamini and Hochberg method [94], [95]) with the *EMMA2* software [96]. The differentially regulated gene probes were automatically annotated, using the corresponding IMGAG gene annotation (http://www.medicago.org/genome/IMGAG/) [97] when available, as well as the annotation of the best hit in TAIR [98] and Swissprot [99] databases.

### Quantitative RT-PCR analyses

RNA samples (three biological repetitions per sample) were isolated using the SV total RNA extraction kit (Promega) following the manufacturer's procedure and analysed with a Bioanalyser (Agilent Technologies). The absence of genomic DNA contamination was verified by quantitative PCR with primers for the *MtMADS1* gene intron and the *EFD* promoter (Table S8). Reverse transcription was performed on 2 to 3 µg of RNA using the superscript reverse transcriptase II (Invitrogen) and anchored oligo(dT). Quantitative RT-PCR analyses were conducted on 384-well plates, using a LightCycler 480 (Roche) with the manufacturer's recommended conditions and the primers shown in Table S8. Cycling conditions were as follows: 95°C for 5 min, 45 cycles at 95°C for 15 s and 60°C for 1 min. We used an ubiquitin gene as an internal standard for sample comparisons.

### *In situ* hybridizations

Four independent experiments were conducted using digoxigenin-labeled riboprobes produced by in vitro transcription with either T7 or SP6 RNA polymerase (antisense and sense probes respectively) (Promega), following the manufacturer's recommendations. Alkaline hydrolysis was carried out in sodium carbonate buffer, pH 10.2 to obtain an average probe length of about 150 nucleotides. Nodules were fixed in 4% paraformaldehyde in 100 mM phosphate buffer (pH 7.4), embedded in paraffin and cut in 8 µm sections, using a Reichert-Jung 2040 microtome. Hybridization was done overnight at 45°C in 4xSSC pH 7.0, 50% formamide, 10% dextrane sulphate, 250 µg.mL^−1^ salmon sperm DNA, 250 µg.mL^−1^ tRNA, 2x Denhardt, and followed by washes once in 4x SSC (5 min room temperature), twice in 2x SSC (30 min at 40°C), once in 1x SSC (60 min, 45°C) and twice in 0.2X SSC (30 min, 45°C). Hybridised probes were detected with anti-digoxigenin antibodies (Roche) bound to alkaline phosphatase, revealed by a standard NBT-BCIP reaction. The revelation of alkaline phosphatase activity was stopped with distilled water (5 min) and background staining was removed by 90 sec washes in 40%, 70%, 100% ethanol, then 70%, 40% ethanol and finally pure water.

### Use of MapMan, LEGoo and the *Medicago truncatula* gene atlas

MapMan software (version 3.5.1) [100], [101], [102] was obtained from http://mapman.gabipd.org/web/guest/mapman and used as recommended. The gene descriptions, given in Table S9, were directly exported from MapMan representations.

The correspondence between Mt16KPlus or Affymetrix gene probes, gene identifiers, published mRNAs and MtGI TC numbers was established with the LEGoo portal (http://www.legoo.org/), using the “Nickname” tool with default settings. When no direct correspondence was found between a Mt16KPlus reporter and an Affymetrix gene probe, the corresponding TC identifier (MtGI9) was first searched and used to look for an Affymetrix probe correspondence. Overall correspondence with a single Affymetrix gene probe was found with ∼93% of the Mt16KPlus reporters. “Nickname” was also used to identify the best hits in TAIR and Swissprot databases.

## Supporting Information

Figure S1 **Example of a WRKY transcription factor gene from the exo1 regulation class activated both in nodules and pathogenic conditions.** Gene up-regulated in wild type and mutant nodules ([19] [20], this study) as well as *M. truncatula* plants treated by yeast elicitor [68] or challenged with the bacterial pathogen *Pseudomonas syringae* [103].
(TIFF)

Table S1 **list of 16kPlus reporters scored as differentially regulated in nodules, as compared to non inoculated, nitrogen-starved roots.**
(XLS)

Table S2 **Cross comparison of differentially regulated genes found in different nodule samples compared to non-inoculated roots.**
(XLS)

Table S3 **Examples of multigene families containing root-expressed genes and nodule-associated genes.**
(XLS)

Table S4 **list of putative regulator genes scored as differentially regulated in nodules when compared to non inoculated, nitrogen-starved roots.**
(XLS)

Table S5 **Search of co-regulated transcription factors using the *Medicago truncatula* gene atlas data.**
(XLS)

Table S6 **Use of the Legoo knowledge base to search for possible expression of nodule-associated regulators in other conditions.**
(XLS)

Table S7 **Quantitative RT-PCR analyses in root samples.**
(XLS)

Table S8 **List of primers used for quantitative RT-PCR analyses.**
(DOC)

Table S9 **List of genes classified by MapMan as related to heme (hemoglobin genes), phenylpropanoids and flavonoids.**
(XLS)
