# Supplementary figures and images for: Transcription Reprogramming during Root Nodule Development in Medicago truncatula
Source: PLoS One. 2011 Jan 27;6(1):e16463. doi: 10.1371/journal.pone.0016463 (PMC3029352; doi:10.1371/journal.pone.0016463)

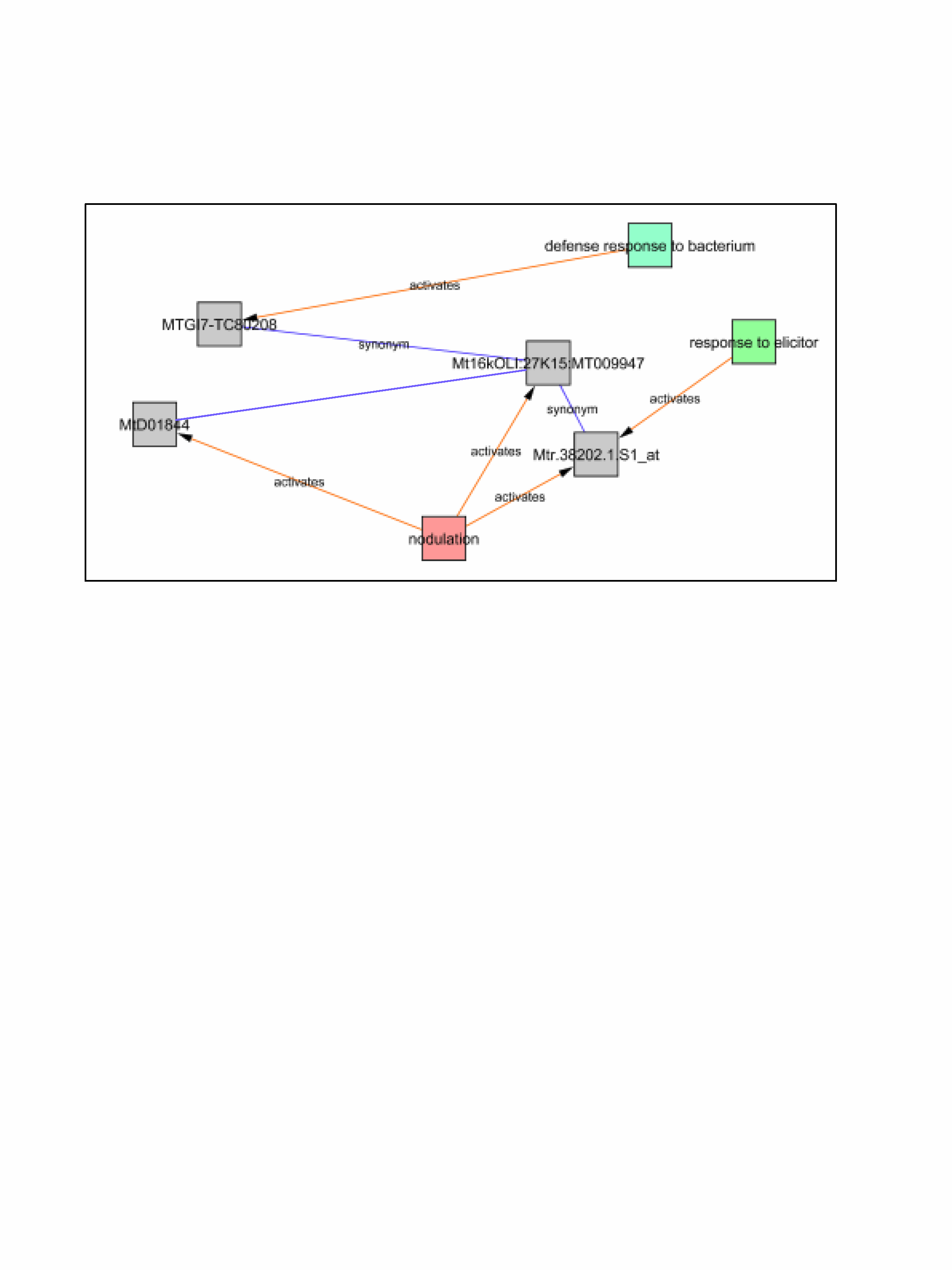

Supplement: Figure S1 — Example of a WRKY transcription factor gene from the exo1 regulation class activated both in nodules and pathogenic conditions. Gene up-regulated in wild type and mutant nodules ([19] [20], this study) as well as M. truncatula plants treated by yeast elicitor [68] or challenged with the bacterial pathogen Pseudomonas syringae [103]. (TIFF) [file pone.0016463.s001.tif]
