# Supplementary material for: Transcription Reprogramming during Root Nodule Development in Medicago truncatula
Source: PLoS One. 2011 Jan 27;6(1):e16463. doi: 10.1371/journal.pone.0016463 (PMC3029352; doi:10.1371/journal.pone.0016463)
Supplement: Table S8 — List of primers used for quantitative RT-PCR analyses. (DOC) [file pone.0016463.s009.doc]

| **Supplemental table 8 Primers used for Q-RT-PCR analyses** | | |
| --- | --- | --- |
| **Identifier** | **Forward** | **Reverse** |
| MT001907 | TCAACTGACAATGCTGCCACAT | GGAGTCCCATTCTTGACGTGATT |
| MT007863 | GGAAAACATTACCAACCCACCA | CAGCATCTGTGTCACACTTCAACA |
| MT007023 | ACAATCCCAACCTAGAGGACCAAT | CGGTAATCTTGGAACATGAGAACC |
| MT001107 | GACCAACCCAAATACACCATCTGT | CAAGATTCACCTCCACTTCCTGAT |
| MT001302 | GTTACGTTGACAATGCGAGAGGA | ACACTCTGGCAACTCTCCGAGA |
| MT006194 | TTCAGCAACAGCAATGGCTTC | GGCATTGAACCAGAGTTTGAGG |
| MT013533 | CAAGAATTAGCCAAGGCGACA | AGCTCCAAATCCACCTTGACC |
| MT004342 | AACGTTTCGCCACATCCAAA | ACCACCGGAACAAAGCTCAAG |
| MT011410 | CTCTATTGCTCTCGGTGCAGCT | CAGAACGTCCTGGAAATGTGTG |
| MT016467 | GTGCTTTCAGAGTGAAGGCAACTT | AAATCCCCACATTGCTTGCA |
| MT008693 | CTGGTTAGAACATGGTGCAGACAC | AGCCCCAAATGCCAAAAAAG |
| MT007813 | ACGCCTTCACTTCTTCATGGTG | CGGTAGTTCTGCGATCCTTGAG |
| MT007526 | AGAGCGATTGGTGAGTTGGCT | CCCGGCAATCATCACGTTAA |
| MT008495 | CATGTTGTGGCCAAGGAAACTT | TCCTCTTTCCCCAAGAGAGTGAG |
| MT007328 | TACACACTCTCCCTCCATTTTCCT | TGAGAAGCCAAAAGCTAAAGCAC |
| MtC70568 | TCCATTGCATGCCTGGAGTT | CCCTCAAACGTACATTGCATCA |
| MtC70235 | CTGGCAATGACATGATGTGCTT | GAGAAGGATCTTCTTGAATGCACC |
| MT003798 | AAAGGTCAGAGAAAGCAGCGC | GGCATGCAAGAAGCGTTTGT |
| MT002234 | GGAATGTAAAGTGTCGCCAGATG | GGATGCAGAAAATGCTCCCATA |
| MT016468 | AGGTGGAGGCTGGTTACCATAAG | GCCTACCACACAAACAACCATCA |
| MT008842 | TCGGATCTACTGTCCACTCTTTGG | TTGGCATGACGATACCGTGTC |
| MT016469 | GCGTGCAATAATTCACCCAAG | GGCAGACTAGCTGAATCTGCAGTA |
| MT015113 | GAGCGAAGAATTGAGCACTGCT | TGCCTTCTTAATTGCAGTTGCC |
| MT000633 | TCATTGGACTTCAGAACGACGTC | AACTCGTGCTATGCATGCTGC |
| MT001725 | AATTTGGCTTGGAAAGGTTCAC | GATCGAATAAGTTGGACTCTTCCC |
| MT009947 | CACCTTCAATATCACCCAACCAA | TGTGAGTAAACGGAACCAGCAA |
| MT010767 | AGATTTCAGCAGATGTGGTAGCGT | CCCTTCAAACCACCCAAAGAG |
| MT006735 | GCAAAATCCTTCACCTCAGACAAG | TGCCTCACTGATTTCACTGGC |
| MT007417 | GGTCACAACCAATGGAAGCCT | GGACACATATCTGGTGGACATTCA |
| MT007335 | TGAGTTATTTGCTCCCACACGAG | TGACGAACCCCTGAAGTAGCAG |
| MT003955 | TTGCTCAGAGGTAGCTGGTCAGA | TCCCACCATAACCTGCATTCC |
| MT010452 | TGGAGTTGGCCATCAGCATT | CCCTCCCATCCTGTCCATATAGA |
| MT003118 | CAACGTTATAGAGGTGTACGCCAA | GGTGACGAATTTCAGAGACCCA |
| MT009966 | TTGTGAGTCATCGTCACCAAGG | CTTAGCTTGGCATCTATTCTTGGC |
| MT008554 | AGTCATGCAGCCTGAGGAGTAGAG | GCACCATTTCCCTCAGCAACT |
| MT001831 | AGCTGTAGTGCAAGAAGGTTCAGA | CGCTACTGTTGACTCCAAATGG |
| MT001896 | CGAAAACAAACTTCCCTTTCCC | ATTCAACGGTGCTGCTTTGG |
| MT008018 | ATGCCTCTACACATGAGCCAGAT | CTTTAGCATCGGTTTTCAACCC |
| MT001094 | ACCCTTTTGCCCCTCAAATTC | AAACACATCTTGCCAACCCG |
| MT007524 | AGCGGATTAGTGTGGAGCAGAA | CCTCACAGGCTATCATGTTGACAA |
| MT014087 | CTCGTTCTGCAGGACAAAGTGTAA | GAAAGCTTGTGCAATGACAGCA |
| MT013931 | GTGCTTCGATCTTTGATGCCTT | CAATAATAGAAGCTTGGTCACCCC |
| MT000483 | ATGCAGTGTCACCAGAGATTTTCC | TATTGACTCCACCATGCCATGTAG |
| MT004882 | TCTCCGATCCAGCATTTGGT | TATCACGCAGCAGGCTCTGTAC |
| MT000406 | GTACAGGTGTCAATCGCATGCT | TGCAAGATCAGATTGGAACCAC |
| MT002230 | GGACAAGAACAGACCAACGAGG | GCTTCTGGTGGAACATCAACAAC |
| MT009912 | GTGGATTTTGTTACAACCCAACG | GAACATCTCTGGATCGGACACA |
| MT011401 | AAGGCCCTCCCTCCTAGAAAAA | GGTGCAGAGACTCCTTGCTTCA |
| MT012940 | GCAGGCAATCACAGAGTGTGAA | TTAGCCCCTGAAGTGTTTCCG |
| MT016301 | AATCCTCCGGTATTTCCTGTGG | TCATGCTGCTGCTAGTACTGCTCT |
| MT007674 | TCTCCCATGGCTTTCAATGC | TTGATCGGACGGTAACTCGTG |
| MT002843 | CAAGAAGCGACGTTCAAGTCTCT | CCCTTGGTCTTTTGTGTTGGTT |
| MT014420 | GCTTTGTATGGTGGTCTCGAGC | GCTTGATGCGACTTTCCAACA |
| MT002996 | CCACTTATGCAGCTGGTTTAGAGA | CTTGGTTCCCAAAGCTGCTATT |
| MT015342 | TCTGCAACAGTCTTCATGGTGGT | CAACCCTCTCAACGTCCAACAA |
| MT004761 | CGGCTGGAAATAGATCAAATGG | CATGCTACTTCCCAAATTCGGA |
| MT016319 | CAGCTCATCTACCAGGACGAACA | CCCATCTTGAGAAGCTTTTTCCTT |
| MT002558 | ATTACTGTGTGATGCTCCGCG | CCATCCTTCTGACTCCGAAACA |
| MT010069 | AATCCTCAGTGGCGAGAATCG | TTGCTGAGAGTAACCACCGGAC |
| MT000081 | GCCTGTTGATCCTTCACCACAT | GGTAAAGAGGCATGAGGCTCAGTA |
| MT000150 | ACCTTTGTTGCCACGCCTTT | AGTGACGTCGAGTTGAGCCAAG |
| MT010098 | GGCAGGCTTTTGAAGACAGTCTT | GCTGCAATGGCTGTTTGGATAT |
| MT009684 | GCTTAGCCAGAGATTCATTGCACT | CTTGTCCATCTTTTTGAGTCCAGG |
| MT009741 | AGAAGTGTAGATGACGGCGCAC | TCTCCGAGGAAGAGCTAAGCTTG |
| MT001976 | CGTTGTATGGCGTGCAATCA | ACCCGCCATTTAAACGTCGT |
| MT007176 | AGCAGATGTACAAAGAGCAGGAGC | AGCTTTGACATGGCTGCATTTC |
| MT016028 | TGGCTAGACTGAGACAAAGTCCAA | CCGTTTCCTCTAGCCCATCAT |
| MT007607 | TACACAACGCAGGAAAACCACA | CTGGAAAAGACTGCTCAAGAAGGA |
| MT016039 | GAAAACGATCAAGTTCCCACTCC | GTGTTTGTTCTGGCACTTCTTCAG |
| MT014860 | CAGACACCAATGGCCCTTTATC | CCAAAGCAGTGGAATTGTGATCT |
| MT016090 | GGTTGAGGATCCGATAACCGA | CACAAACGCCGTTCCTTCAT |
| MT008226 | GCAAAGAGCATGGAAAGTGCAT | GGTTTCACCTCAGCCTCACATTT |
| MT007789 | CGGAGTTTGCTCGTGATATCCT | GCTGCCGTACAAAGCTAGAGAAGT |
| MT000567 | CTCAATGCTGCACAAGGATCG | CCCTAACCCTATGAGCTCTAACCG |
| MT016132 | GGAAACAATGCCATTGAGCC | TTATGGATGTGCGGTTTCTCTTG |
| MT000719 | GTGTGTTGCTGACATGGTGCTC | CAAAGCGTGCTCGTTATCATCA |
| MT010489 | GCTGGACTTGAATTTGACACCTCT | GGAATTGGTGTTGGTGGTGACT |
| MT008661 | TTGGTGCTGAGAGGCTACATTATG | TGCTCTGTCCATGGTGATAACTTC |
| MT006425 | CTTTGCCTCAACTTCCTTCTGCT | CAATCCTGTAGGCCAGATCCAG |
| MT015782 | TCCAAGCTGCACAGTGAAGAA | TGCAACCACAACTGATGGATC |
| MT013054 | AGCAACATTGTTTGGCTCGG | CGACCCTTTCTTTTCCTTCACAC |
| MT015838 | TGAATCGCTTAAGAACGTGGTG | CTCGTCCTTTATATCCTCCAAAGG |
| MT000471 | AGAGGTTTATTGGCGATGGAGAG | CATCAGTCTCACGTCCATTGGA |
| MT003202 | GCATAATGCTGATCCAGAACCC | TCGACCGCAAGTAGAGAACCTC |
| MT011705 | CTGAAGACAAGCAGATCAACCGT | TACACATCTCGTTCCCCTCCTC |
| MT000685 | TATTTGCAGCAGGTGACTGTCG | CCCTCTGAAATAGCCCATACCAC |
| MT016193 | CGAATATCGTCTTGCCAACGTT | AGCACCCAATCATCAAGCCTC |
| MT000284 | CAGTACCTGAAATGTGGTCACCG | CAACTGATCATAAGCTTCTCCCCA |
| MT000919 | CACGACCACGAGGTGGTTTTT | ACCCTCTCCATACCAACCCCTT |
